# New Opportunities in Mid-Infrared Emission Control

**DOI:** 10.3390/s150922724

**Published:** 2015-09-09

**Authors:** Peter Geiser

**Affiliations:** Norsk Elektro Optikk A/S, Prost Stabels vei 22, 2019 Skedsmokorset, Norway; E-Mail: geiser@neo.no; Tel.: +47-6797-4700

**Keywords:** mid-infrared, nitric oxide, sulfur dioxide, quantum cascade laser, interband cascade laser, *in situ*, gas sensing

## Abstract

Tunable laser absorption spectroscopy (TLAS) has been well accepted as a preferred measurement technique for many industrial applications in recent years, especially for *in situ* applications. Previously, mainly near-infrared lasers have been used in TLAS sensors. The advent of compact mid-infrared light sources, like quantum cascade lasers and interband cascade lasers, has made it possible to detect gases with better sensitivity by utilizing fundamental absorption bands and to measure species that do not have any absorption lines in the near-infrared spectral region. This technological advancement has allowed developing new sensors for gases, such as nitric oxide and sulfur dioxide, for industrial applications. Detection limits of better than 1 ppm · m for nitric oxide and better than 10 ppm · m for sulfur dioxide are demonstrated in field experiments.

## 1. Introduction

Since the negative effect of pollutant gases on the environment has become evident, governmental agencies, like the United States Environmental Protection Agency (US EPA) or the Umweltbundesamt (UBA) in Germany, are adjusting emission directives to lower levels on a regular basis. Suppliers of measurement instrumentation are challenged to enhance the performance of sensors at the same time. In particular, nitrogen oxides (NO_x_) and sulfur dioxide (SO_2_) are of great interest, since they are the main precursors of acid rain [1].

The majority of industrial users has accepted near-infrared tunable laser absorption spectroscopy (NIR-TLAS) [2] as the best available technique for *in situ* emission and process control measurements due to easy installation, fast response time, high accuracy and low maintenance requirements of the sensors. Despite the huge success of NIR-TLAS sensors, there are a few limitations: for some important gases, the NIR absorption line strengths and, thus, the sensitivities of sensors are too low (e.g., nitric oxide, NO), and some species cannot be measured at all, since they do not have any absorption features in the near-infrared (e.g., sulfur dioxide).

Fundamental absorption bands in the mid-infrared spectral region (MIR), the so-called fingerprint region between 3 µm and 12 µm, are orders of magnitude stronger than the overtone and combination bands in the near-infrared, which makes the mid-infrared spectral region predestined for highly sensitive and selective measurements. However, for a long time, MIR laser sources were bulky and in many cases unreliable, which precluded them from being used in almost all industrial applications. Fortunately, recent progress in quantum cascade lasers (QCLs) (e.g., [3,4,5]) and interband cascade lasers (ICLs) (e.g., [6,7]) made compact and reliable light sources available that are suitable for *in situ* measurements even in harsh environments.

In this paper, applications for mid-infrared sensors of nitric oxide and sulfur dioxide are described. The sensors are based on the commercial LaserGas II ™ platform [8]. The sensors are manufactured by Neo Monitors A/S, a subsidiary of Norsk Elektro Optikk A/S (NEO). The sensors consist of a transmitter unit (integrating among others, a laser source, collimation optics and driver electronics) and a receiver unit with built-in detector and read-out electronics. Single-line absorption spectroscopy and, in particular, wavelength modulation spectroscopy (WMS) are used as the detection scheme. This technique has proven to be very powerful in trace gas sensing due to the ability of performing interference-free measurements directly in the process or across stacks without preconditioning and sample extraction.

The temperature of the laser is stabilized with high accuracy (typically in the mK range) to set the emission wavelength of the light source. A direct current (DC) is applied to operate the laser above its threshold, and a current ramp is used to tune the wavelength over an absorption feature of the targeted molecule. In order to improve the signal-to-noise ratio, a sinusoidal waveform is added on top of the current ramp. On the receiver side, the second harmonic component of the detector signal is extracted using a hardware mixer. The second harmonic signal is sampled, and 64 points of the whole wavelength scan are recorded. Adjustable low-pass filters embedded in the signal-processing software are used to further improve the signal-to-noise ratio. More detailed descriptions about the principles can be found, for example, in Linnerud *et al.* [2] and Kluczynski *et al.* [9].

In emission control applications, the investigated gas mixtures consist mainly of water vapor, carbon dioxide and nitrogen, typically at the percent level. Other gases, like nitrogen oxides, carbon monoxide and methane, are typically in at ppm level range. Depending on the application, also other gases, such as ammonia or sulfur dioxide, can be present. Table 1 gives an overview of a generic gas composition that is used for the following analysis and line selection. In emission control, the gas temperatures can vary between room temperature and 400 °C; the pressure is typically around the ambient one; and the measurement path length can be between a few tens of centimeters to several meters.

**Figure 1:**
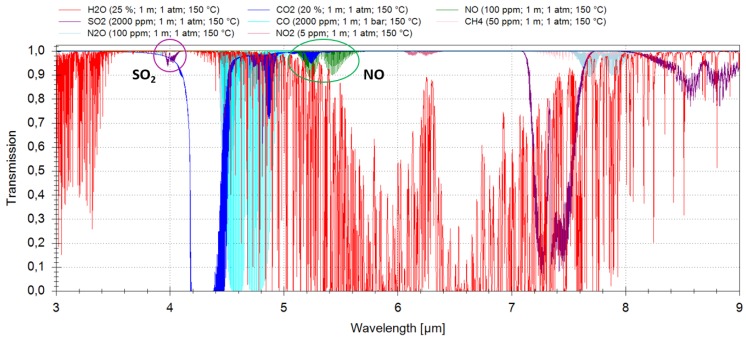
Simulation of a transmission spectrum of a typical gas mix in emission control using the HITRAN and HITEMP databases.

Databases like HITRAN (high-resolution transmission molecular absorption database) [10] and HITEMP (high-temperature molecular spectroscopic database) [11] were used to select suitable absorption lines for nitric oxide and sulfur dioxide that are interference-free from other molecules present in the gas mixture. Figure 1 depicts a simulated transmission spectrum between 3 µm and 9 µm. In addition to the above listed gas composition, 2000 ppm sulfur dioxide were added to the gas mix, as well. In the simulation, it was assumed that the optical path length (OPL) is 1 m, the pressure the ambient one and the gas temperature 150 °C.

**Table 1:** Overview of a typical gas mixture in emission control applications. Depending on the application, traces of other gases can be present, as well. The table was generated using input from customer inquiries.

Species	Chemical Formula	Typical Concentration
Water vapor	H_2_O	10%–25%
Carbon dioxide	CO_2_	10%–20%
Nitric oxide	NO	0–100 ppm
Nitrogen dioxide	NO_2_	0–5 ppm
Nitrous oxide	N_2_O	0–100 ppm
Methane	CH_4_	0–50 ppm
Carbon monoxide	CO	0–2000 ppm
Nitrogen	N_2_	rest

## 2. Sulfur Dioxide

As shown in Figure 1, three absorption bands of sulfur dioxide can be considered for typical *in situ* emission monitoring applications: around 4 µm, around 7.4 µm and around 8.7 µm. For all three wavelength regions, quantum cascade lasers are available; for the first absorption band, interband cascade lasers are available, as well. Due to their superior thermal characteristics and the possibility to integrate ICLs in compact TO66-cans instead of more bulky high heat load (HHL)-packages, like those typically used for QCLs, it was decided to use an ICL as the light source for the sulfur dioxide sensor.

The two absorption bands around 7.4 µm and around 8.7 µm are significantly stronger than the absorption band around 4 µm, and measurements would thus be more sensitive. However, in emission monitoring, where fuel containing sulfur is combusted (e.g., in coal fire plants), the concentration level of SO${}_{2}$ can vary between 50 ppm and 5000 ppm, so that low ppm sensitivities, achievable with QCLs at 7.4 µm [12], are not necessarily required. Therefore, the 4-µm wavelength region is the appropriate choice.

Detailed software simulations have led to a selection of interference-free SO_2_ absorption lines, and nanoplus Nanosystems and Technologies GmbH has developed a mono-mode distributed feedback (DFB) ICL for one of these wavelengths [7]. The laser has a maximum output power of about 4 mW (driving conditions: 70 mA, ~5 V), a current tuning rate of 0.11 cm^−1^/mA and a temperature tuning rate of 0.24 cm^−1^/K. The laser was integrated in a modified transmitter unit of a LaserGas II ^™^ sensor. The laser beam is collimated and focused using antireflection (AR)-coated CaF_2_-lenses with a focal length of about 75 mm. An InAsSb-detector (Hamamatsu P11120-201) is used in the receiver unit.

**Figure 2:**
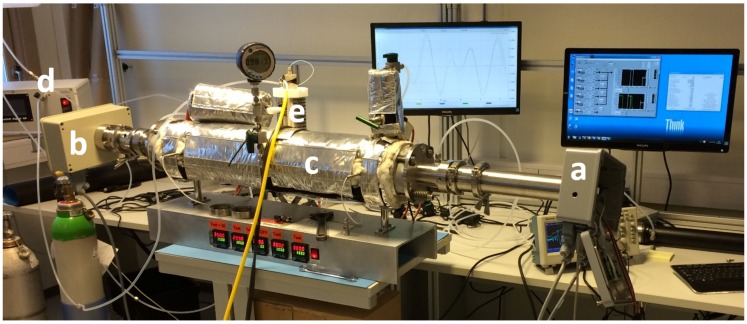
Sulfur dioxide sensor mounted on home-made heated cell: (**a**) transmitter unit; (**b**) receiver unit; (**c**) heated cell; (**d**) gas mixing system and (**e**) evaporator.

In order to characterize the sensor, it was mounted onto a home-made heated cell with an optical path length of 70 cm and gas temperatures up to 400 °C (Figure 2). The sensor was calibrated with a known concentration of sulfur dioxide.

**Figure 3:**
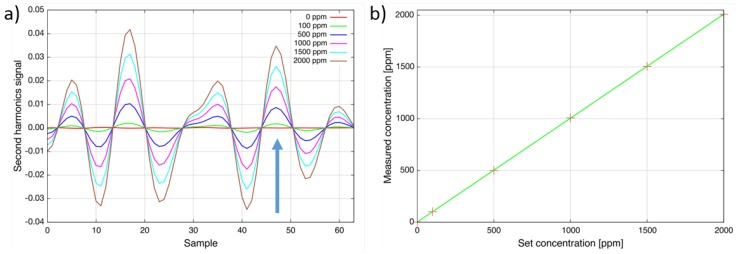
(**a**) Second harmonic spectra of sulfur dioxide for different concentration levels at 160 °C. The target line at Sample 47 is indicated by an arrow. The total wavelength tuning is approximately 1 cm^−1^; (**b**) Linear response at 160 °C. These figures have previously been used in the presentation of Geiser *et al.* [13].

To verify the linearity of the sensor response, the heated-cell (T = 160 °C) was filled with different concentration levels of SO_2_ from 0 ppm up to 2000 ppm (Figure 3a). Figure 3b compares the set concentration at the gas mixing system with the measured concentration. As can be seen, the response of the sensor is linear within the accuracy of the gas mixer and the sulfur dioxide sensor noise level [13].

Initially, the absorption line at Sample 17 was selected, and the lower detection limit of the sensor was determined to be around 3 ppm (2 × 10^−5^ (rel. abs.)). Due to an unexpected strong interference with nitrous oxide, the line at Sample 47 was used in the following instead. Furthermore, modulation amplitude and software filter settings were changed to minimize the interference. The lower detection limit has increased to 10 ppm (7 × 10^−5^ (rel. abs.)). The dynamic range reaches up to 1 % · m. The gas temperature can be up to 300 °C. This makes the sensor ideal for emission and even process control applications.

**Figure 4:**
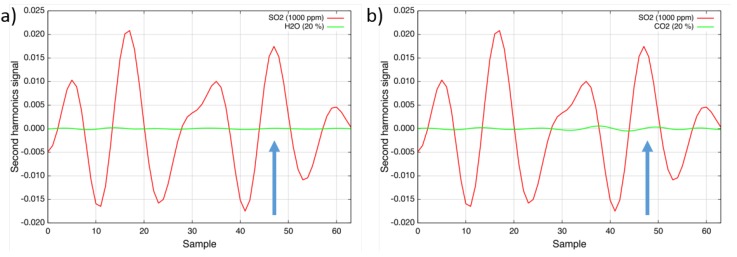
(**a**) Second harmonic spectra of 1000 ppm sulfur dioxide (red) and 20% water vapor (green) at 160 °C; (**b**) Second harmonic spectra of 1000 ppm sulfur dioxide (red) and 20% carbon dioxide (green) at 160 °C.

To determine the interference of other gases at typical emission control levels, the absorption cell was filled with different species. As can be seen in Figure 4a, the measurement is interference-free from water-vapor; levels of up to 30% do not have any influence on the measured concentration level. The interference from carbon dioxide (20%) was determined to be ±2 ppm · m (Figure 4b). Ammonia (NH_3_), methane (CH_4_) and nitrous oxide (N_2_O) in typical emission control application concentrations interfere only at sub-ppm levels. Other gases, like carbon monoxide (CO), nitric oxide (NO) and nitrogen dioxide (NO_2_), do not absorb in the investigated wavelength region [10] and, therefore, do not have any influence on the sulfur dioxide measurement.

**Figure 5:**
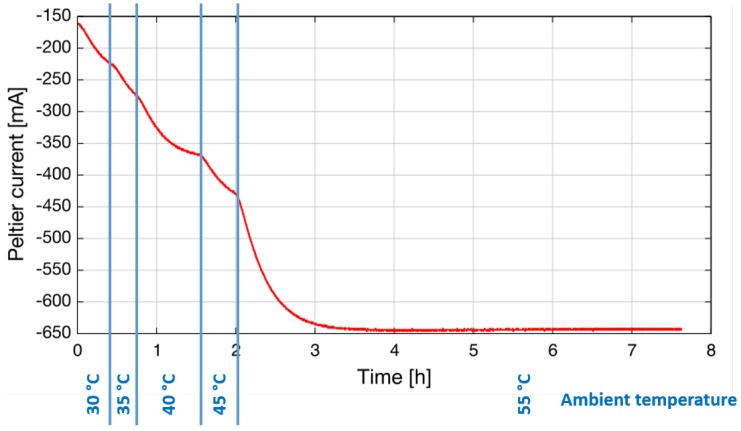
During the climate chamber test, the Peltier current that is necessary to stabilize the laser temperature was logged for different ambient temperatures, as indicated in blue at the bottom.

An important requirement of sensors for industrial applications is the ability to operate at a wide range of ambient temperatures from arctic to tropical conditions. Climate chambers allow the simulation of different ambient temperatures in the laboratory. For this test, the sensor was mounted inside a climate chamber, and the Peltier current that is necessary to stabilize the laser temperature (here: T_op_ = 17.5 °C) was logged. The ambient temperature was increased in several steps from 30 °C–55 °C. As can be seen in Figure 5, it is possible to stabilize the laser temperature using about 650 mA at an ambient temperature of 55 °C. This is approximately 50% of the maximum allowed current to the Peltier.

It is important to emphasize that no external cooling (air purge or water cooling) is required, which is an important difference from QCL-based systems that still require this kind of cooling in hotter environments (see Section 3).

The first application was identified at Carbon Service & Consulting GmbH & Co. KG in Germany: activated charcoal with high ammonia and sulfur content is recycled by heating it in an oven. The sulfur desorbs and can be converted to sulfur dioxide by adding oxygen. The SO${}_{2}$ is later filtered out or converted again using a catalyzer. A SO${}_{2}$ sensor is required to regulate and protect the downstream filter from a too high concentration and, thus, fatal damage.

**Figure 6:**
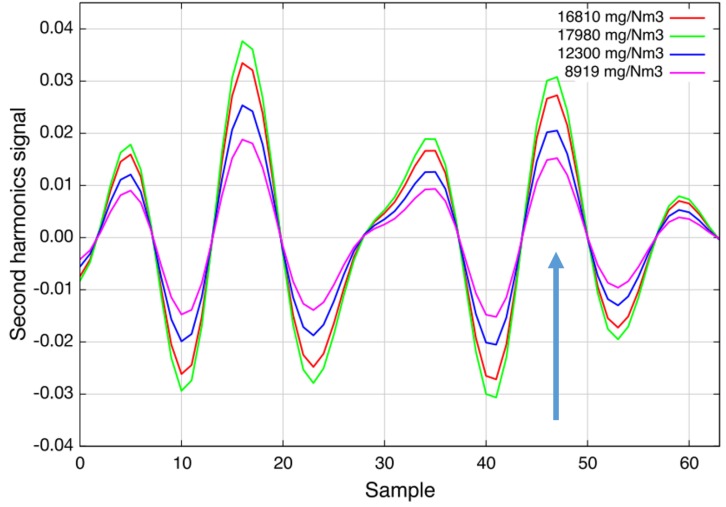
Second harmonic spectra of sulfur dioxide during a cleaning process of heavily-contaminated activated charcoal. This figure was previously used in the presentation of Geiser *et al.* [13].

Figure 6 shows the second harmonic spectra recorded during a recycling process. The measured concentrations are in the range from 9 g/Nm${}^{3}$ to 18 g/Nm${}^{3}$ and confirm the expectations of the plant operator [13]. The sensor was installed in April 2015 and has worked since then without any complications. More results will be presented elsewhere at a later stage.

## 3. Nitric Oxide

In many cases, near-infrared nitric oxide sensors do not meet the requirements of industrial applications: either the sensitivity is too low, interference with water vapor too strong (especially above 300 °C) or both.

Again, software simulations based on the HITRAN [10] and HITEMP [11] databases have led to a selection of interference-free absorption lines of nitric oxide in the fundamental absorption band around 5.3 µm in a typical industrial gas mixture (Table 1 and Figure 1). Since ICLs with sufficient output power and operation temperature well above ambient temperature are not yet commercially available, a QCL was chosen instead. In cooperation with Corning Incorporated (now Thorlabs Quantum Electronics, Inc., Dearborn Heights, MI, USA), a quantum cascade laser chip for one of these lines was prepared [14] and integrated into an optimized HHL-package [12]. The laser has a maximum output power of about 30 mW (driving conditions: 230 mA, ~10.5 V), a current tuning rate of 0.02 cm${}^{-1}$/mA and a temperature tuning rate of 0.12 cm${}^{-1}$/K. The QCL was implemented in a spectrometer setup and first tested in the lab with the above home-made heated cell (same as shown in Figure 2).

**Figure 7:**
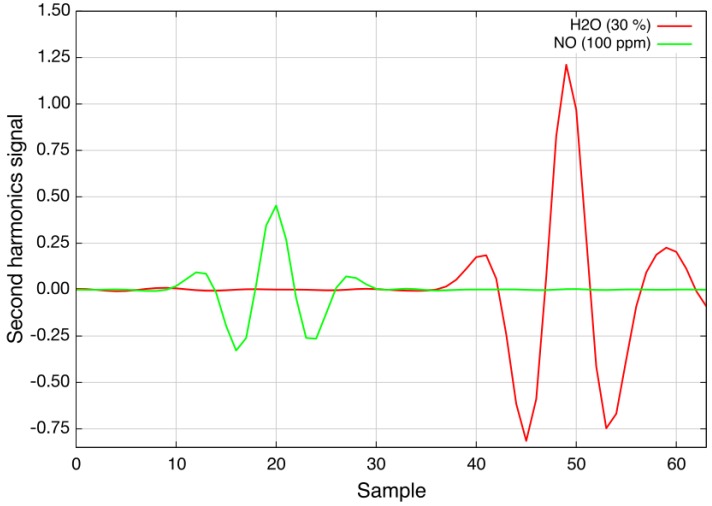
Second harmonic spectra of 30% water vapor (red) and 100 ppm nitric oxide (green) at 400 °C (OPL = 70 cm, p = ambient). The total wavelength tuning is approximately 0.6 cm${}^{-1}$.

The measurements showed good agreement with the previously-performed simulations, and the sensitivity of the spectrometer was determined to be around 4 ×10${}^{-4}$(rel. abs.), which leads to a lower detection limit of better than 1 ppm · m at 400 °C. Figure 7 shows second harmonic spectra recorded at 400 °C and an optical path length of 70 cm: the green trace depicts 100 ppm nitric oxide and the red trace 30% water vapor.

The measurement is interference-free from water vapor concentrations of up to 30% and carbon dioxide (CO${}_{2}$) of up to 20% at an optical path length of 2 m. Other gases, like ammonia (NH${}_{3}$), methane (CH${}_{4}$) or nitrous oxide (N${}_{2}$O), at typical combustion concentration levels do not influence the nitric oxide measurement either.

**Figure 8:**
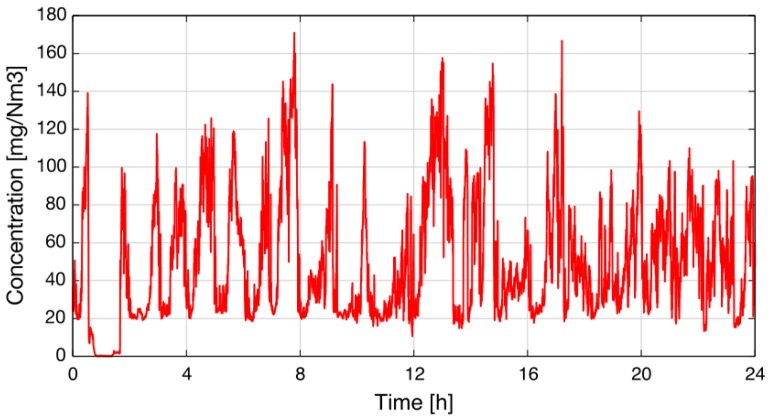
Concentration recorded over 24 hours at Elkem AS in Salten (Norway) [15].

After the tests in the lab, the sensor was installed in a silicon plant of Elkem AS in Salten (Norway) to demonstrate the sensor’s feasibility. The sensor was mounted on a duct with a diameter of 300 cm, and insertion tubes were used to shorten the absorption path length to 80 cm. The gas streaming through the duct had a temperature of about 270 °C. The measured concentration was logged for one day, and the result is depicted in Figure 8 [15].

**Figure 9:**
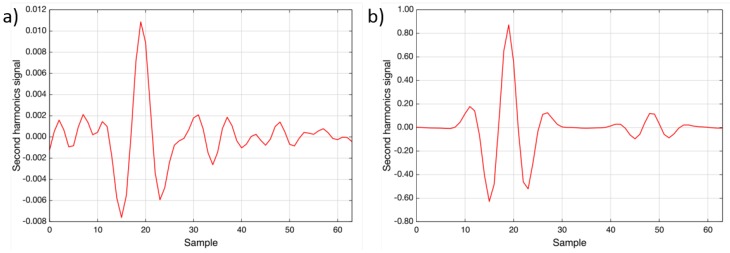
Two recorded spectra during the measurement campaign: (**a**) was recorded during the restart after power failure (1.5 mg/Nm${}^{3}$ NO); (**b**) was measured during regular operation (114.8 mg/Nm${}^{3}$ NO) [15].

A power failure of the plant occurred at the beginning of the measurement. All ducts were purged with ambient air, and therefore, the NO concentration dropped to zero. Figure 9a shows a spectrum recorded during the restart of the plant. From the measured concentration (1.5 mg/Nm${}^{3}$) and the noise level, a detection limit of better than 1 ppm · m can be calculated, which confirms the laboratory result. Figure 9b shows a spectrum recorded during regular operation. The concentration was determined to be 114.8 mg/Nm${}^{3}$; also, the water vapor line is visible at Sample 49 [15].

According to Kamfjord [16], the shape of the concentration measurement in Figure 8 is as expected: after refilling the furnace with raw material, the concentration drops to a base-level of about 20 mg/Nm${}^{3}$ (30 ppm at 270 °C) because of chemical reactions influencing the NO formation at the furnace surface. After a while, these reactions end, and the NO concentration rises slowly at the surface; typically, NO concentrations of 120–140 mg/Nm${}^{3}$ (180 ppm–210 ppm at 270 °C) are emitted. Afterward, the cycle starts from the beginning when new material is added.

**Figure 10:**
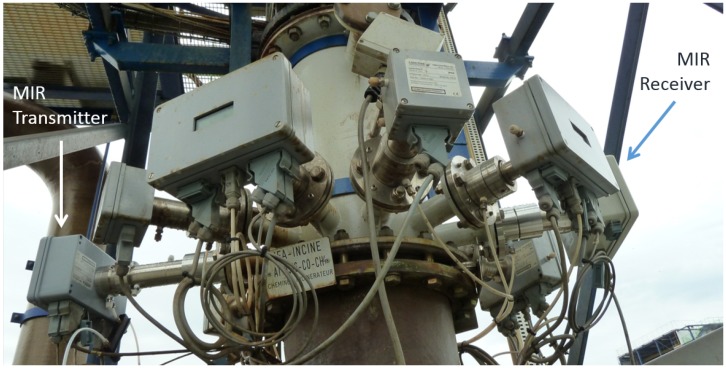
Installation of the mid-infrared NO sensor on a stack of an Arkema Forane-plant near Lyon (France). This photograph has previously been used in the presentation of Kaspersen *et al.* [17].

Finally, the NO sensor was mounted at an Arkema Forane-plant where it replaced an NIR NO sensor that was not able to meet the required detection limit. Figure 10 shows the MIR sensor amidst several NIR sensors for other gases [17]. In this installation, the sensor had to be purged with ambient air to maintain its operational temperature. Purge air is usually available at industrial sites, so this does not constitute a problem.

**Figure 11:**
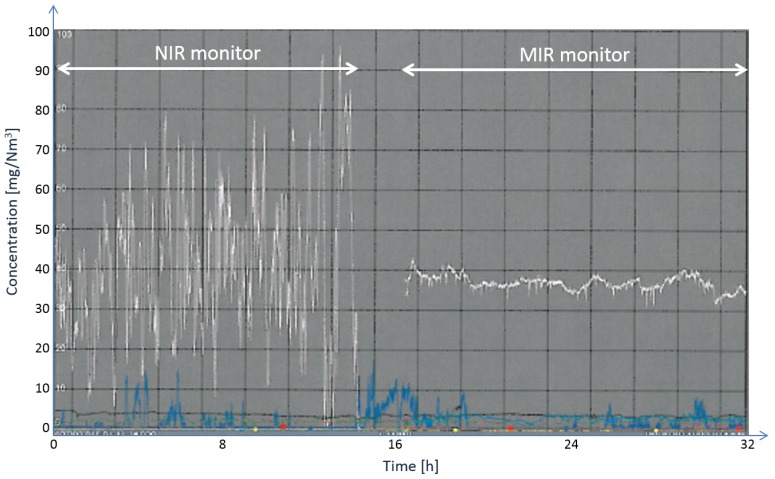
Nitric oxide concentration readings (white trace) with an NIR sensor (**left**) and an MIR sensor (**right**) at the same measurement point [17]. The blue trace depicts concentration readings of an NIR nitrogen dioxide (NO${}_{2}$) sensor.

Figure 11 shows a comparison of the NO concentration readings with the NIR sensor on the left-hand side and the MIR sensor on the right-hand side [17]. A clear improvement can be observed. The sensor was installed in June 2012 and has been running since then (until the time of preparing this manuscript) without any problems or signs of degradation of performance.

Selective non-catalytic reduction (SNCR) [18] and selective catalytic reduction (SCR) [19] are methods to reduce NO${}_{x}$ emissions from combustion processes. In such a deNOx-process, either gaseous ammonia or urea is used to convert NO${}_{x}$ and NH${}_{3}$ into water vapor and nitrogen (N${}_{2}$). The amount of ammonia needed depends on the NO${}_{x}$ content of the process gas. Especially in a dynamic process where the fuel composition changes rapidly, the variation of the NO concentration can be quite high. To regulate the deNOx-process and, thus, the amount of ammonia added to the process gas, the NO${}_{x}$ content has to be measured *in situ*, very accurately, and with a fast time response as close as possible to the combustion zone. Typically the amount of non-converted ammonia is measured to control a deNOx-process (“ammonia slip”), since NIR NO measurements usually suffer from severe water vapor interference at gas temperature above 300 °C. Industrial users, however, would like to measure the nitric oxide concentration directly in the raw gas, meaning as close as possible to the combustion process before the gas is filtered; *i.e.*, the process gas has a high dust load and contains many other gas components, as well.

Recently, a NO sensor was installed at an incinerator for household waste in Germany. Since the composition of household waste can vary a lot, the nitric oxide concentration can also vary significantly on a short time scale.

**Figure 12:**
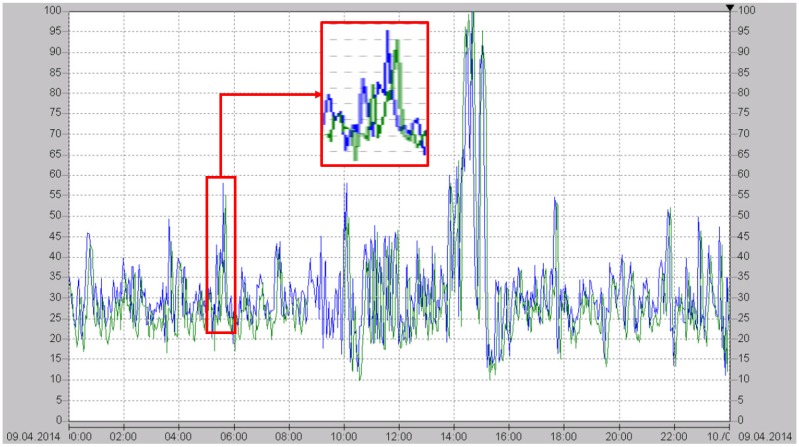
Twenty-four-hour comparison plot of the presented MIR NO sensor (blue) measuring the raw gas close to the combustion process and the final emission measurement of the clean gas at the chimney (green) [20]. Both concentrations are expressed as mg/m${}^{3}$. The inset shows the concentration readings between 5:00 and 6:00 in more detail.

Figure 12 shows a 24-hour plot of an emission monitoring measurement of the clean gas at the chimney (green) and the *in situ* TLAS NO measurement of the raw gas (blue) [20]. As can be seen, the TLAS signal is available significantly earlier than the emission monitoring signal, so that the deNOx-regulator can respond much more quickly to changes in the NO concentration. This allows a deNOx-regulation with a much higher efficiency than is possible with an ammonia-based regulation.

## 4. Conclusions

In the above presented work, it was demonstrated that mid-infrared gas sensing is a very powerful tool for emission and process control applications where *in situ* measurements are required. The two most sophisticated and compact mid-infrared laser source types were used in the presented sensors, and they both have proven to be suitable for field applications. Although the price level of sensors based on a quantum cascade laser are considered to be high (compared to UV and NIR absorption sensors) and, therefore, not attractive for end-users, the advantages of such a system outweigh that according to industrial users’ feedback. Interband cascade lasers are currently significantly less expensive than quantum cascade lasers, and therefore, the overall sensor system price will be soon at a similar level as well-accepted NIR-TLAS sensors for *in situ* applications. The long-term stability and reliability of MIR sensors are as good as their NIR counterparts, and now, the way is open for new exciting applications that were impossible until recently.
